# An Efficient and Footprint-Free Protocol for the Transdifferentiation of Hepatocytes Into Insulin-Producing Cells With IVT mRNAs

**DOI:** 10.3389/fgene.2020.00575

**Published:** 2020-06-05

**Authors:** Shinan Ma, Mengjie Yang, Wenhui Zhou, Longjun Dai, Yan Ding, Xingrong Guo, Yahong Yuan, Junming Tang, Dongsheng Li, Xiaoli Wang

**Affiliations:** ^1^Hubei Key Laboratory of Embryonic Stem Cell Research, Taihe Hospital, Hubei University of Medicine, Shiyan, China; ^2^Department of Medical, Southeast University, Nanjing, China; ^3^Department of Neurosurgery, Taihe Hospital, Hubei University of Medicine, Shiyan, China; ^4^Department of Surgery, University of British Columbia, Vancouver, BC, Canada

**Keywords:** transdifferentiation, IVT mRNA, TGIF2, hepatocytes, insulin-producing cells, transcription factors

## Abstract

**Background:**

Direct transdifferentiation of adult somatic cells into insulin-producing cells (IPCs) is a promising approach for cell-based therapies for type 1 diabetes mellitus. Liver cells are an ideal source for generating IPCs because they have regenerative ability and a developmental process similar to that of the pancreas. Pancreas versus liver fate is regulated by TALE homeoprotein (TGIF2) during development. Here, we wanted to investigate whether TGIF2 could enhance the efficiency of transdifferentiation of hepatocytes into IPCs induced by three pancreatic transcription factors (pTFs), i.e., Pdx1, NeuroD, and Mafa, which are crucial for pancreatic development in the embryo.

**Methods:**

The *in vitro* transcribed (IVT) mRNAs of TGIF2 and the three pTFs were synthesized *in vitro* and sequentially supplemented in hepatocytes. On day 6, the expression of transcription factors was assessed by quantitative real-time polymerase chain reaction (qRT-PCR), and insulin expression was detected by immunofluorescence. Glucose-stimulated insulin secretion was assessed by enzyme-linked immunosorbent assay (ELISA). The key genes controlling cell polarity and the Wnt/PCP signaling pathway were assayed by qRT-PCR, and the level of JNK protein phosphorylation, which regulates the Wnt/PCP signaling pathway, was detected by western blotting.

**Results:**

IVT mRNAs could be efficiently transfected into hepatocytes. Quantitative real-time polymerase chain reaction results revealed that compared with ectopic expression of the three pTFs alone, ectopic expression of the three pTFs plus TGIF2 could strongly reduce hepatic gene expression and subsequently improve the induction of a set of pancreatic genes. Immunofluorescence analysis showed that TGIF2 expression could double the transdifferentiation yield; 30% of the cells were insulin positive if induced by TGIF2 plus the 3 pTFs, while only 15% of the cells were insulin positive if induced by the three pTFs alone. ELISA analysis confirmed that glucose-stimulated insulin secretion was less efficient after transfection with the three pTFs alone. The differentiated cells derived from the addition of TGIF2 mRNA could form islet-like clusters. By contrast, the cells differentiated with the three pTFs did not form clusters under the same conditions. Tgif2 induced transdifferentiation more efficiently by remodeling the expression of genes in the Wnt/PCP pathway. Overexpression of TGIF2 in hepatocytes could activate the expression of key genes controlling cell polarity and genes in the Wnt/PCP signaling pathway, increasing the level of JNK protein phosphorylation.

**Conclusions:**

Our study established a novel footprint-free protocol for efficient transdifferentiation of hepatocytes into IPCs using IVT mRNAs of TGIF2 and 3 pTFs, which paved the way toward a clinical application.

## Background

Type 1 diabetes mellitus (T1D) is an autoimmune-mediated disease that is characterized by pancreatic beta cells being attacked by the immune system. The primary therapy is the injection of exogenous insulin to normalize blood glucose levels, but this is often accompanied by diabetic complications. A preferable treatment is transplantation of pancreatic islets. Unfortunately, the limited supply of donor islets and the requirement for life-long immune suppression make this approach impractical for large-scale application. This hurdle has led researchers to seek new alternative therapies (Omami et al., 2017; Nevo-Shenker et al., 2019). Cell-based therapy has opened a new corridor for T1D therapy, as beta-like cells or insulin-producing cells (IPCs) have been successfully induced in laboratories (Kopan et al., 2018; Espona-Noguera and Ciriza, 2019).

Direct transdifferentiation of some somatic cells into other functioning cell types is a novel approach for cell-based therapies (Heathman et al., 2015; Allickson, 2017). Recently, transcription factor-mediated transdifferentiation has successfully regenerated beta-like cells or IPCs from other types of cell sources (Vieira et al., 2017; Amirruddin et al., 2019; Cierpka-Kmiec et al., 2019). Hepatocytes are first on the list of cell sources with the potential to be transdifferentiated into IPCs, as the early developmental processes of the islets and the liver are exactly the same until the bud emerges. The liver also expresses a variety of transcription factors related to pancreatic development and has strong regenerative capacity (Meivar-Levy and Ferber, 2015; Meivar-Levy and Ferber, 2019). Therefore, the use of liver cells to obtain IPCs has broader clinical application prospects. A number of previous studies showed that hepatocytes could be reprogrammed into IPCs by pancreatic transcription factors (pTFs). Among these pTFs, pancreatic duodenal homeobox-1 (PDX1), neuronal differentiation 1 (NeuroD1), and MAFbZIP transcription factor A (Mafa) are the most extensively explored (Zhu et al., 2017). Pancreatic duodenal homeobox-1 is a key transcription factor involved in pancreatic embryogenesis. Pancreatic duodenal homeobox-1 is highly expressed in the early stage of pancreatic development and participates in the development, differentiation, maturation, and proliferation of pancreatic islet cells. Neuronal differentiation 1 plays a crucial role in the middle stage of pancreatic development, controlling the transformation of cells into pancreatic endocrine precursor cells. Mafa participates in late development of the pancreas, inducing further maturation of pancreatic endocrine precursor cells into islet beta cells (Kaneto et al., 2009). Pancreatic transcription factors are usually delivered by vectors such as plasmids or viruses. These delivery methods cannot accurately modulate the expression of transcription factors, and the obtained cells have some potential biosafety hazards that mean they cannot be used for follow-up clinical research. The use of *in vitro* transcribed (IVT) mRNA has several advantages in the regulation of transdifferentiation. It does not need to reach the nucleus to be functional and does not integrate into the genome, which means it has no risk of insertion mutagenesis. Protein expression can also be controlled accurately by adding IVT mRNAs at different times and dosages (Ida et al., 2018). These characteristics make IVT mRNAs very convenient and safe for future clinical use. In our previous work, we successfully differentiated human umbilical cord mesenchymal stem cells into IPCs with PDX1 mRNA (Wang et al., 2014).

Although the sequential introduction of three pTFs could improve the efficiency of hepatocyte transdifferentiation into IPCs, this efficiency was consistently limited to <15% (Berneman-Zeitouni et al., 2014). The presence of antagonists in hepatocytes may limit the plasticity of cells and hinder the transformation of hepatocytes into IPCs. Some regulators of pancreas and liver development may take part in decisions regarding cellular plasticity transitions. It was found that triple amino acid ring expansion homologous frame TGIF2 (TGIF2) in endodermal cells may be a crucial developmental regulator in deciding pancreas versus liver fate. TGIF2 ectopically expressed in hepatocytes could suppress hepatic transcriptional expression and initiate transcription of a subset of pancreatic genes. This TGIF2-dependent fate selection mechanism controls the generation of pancreatic progenitors and requires further investigation in terms of cellular identity and plasticity (Cerda-Esteban et al., 2017).

In this study, we aimed to establish an efficient and footprint-free way to transdifferentiate hepatocytes into IPCs via the combined use of IVT mRNAs for TGIF2, PDX1, NeuroD1, and Mafa.

## Materials and Methods

### Isolation and Culture of Mouse Hepatocytes

This experiment was performed in compliance with the relevant Chinese regulations and approved by the Hubei University of Medicine Animal Ethics Committee. A two-step collagenase perfusion method was used to isolate hepatocytes from C57/BL6 mice aged 8–10 weeks (Nagasaki et al., 2014). Inhalation of 2% isoflurane was performed to anesthetize the mice, and then the abdominal cavity was opened to reveal the portal vein. Calcium- and magnesium-free phosphate-buffered saline (PBS) was perfused through the portal vein at 5 mL/min for 5 min and then changed to Dulbecco’s modified Eagle’s medium (DMEM) with 1 mg/mL collagenase II solution at 8 mL/min for approximately 10 min. The entire liver was removed to a petri dish containing DMEM medium at room temperature. The crude hepatocyte suspension was filtered through a gauze mesh filter (100 μm) and centrifuged. The cells were plated at a density of 0.4 × 10^6^ cells/mL and cultured in DMEM supplemented with 10% fetal calf serum, 100 units/mL penicillin, 100 ng/mL streptomycin, 250 ng/mL amphotericin B (Biological Industries), 20 ng/mL EGF and 10 mM nicotinamide (Sigma) at 37°C in a humidified atmosphere with 5% CO_2_ and 95% air.

### *In vitro* Transcribed mRNA Synthesis and Transfection

**FIGURE 1:**
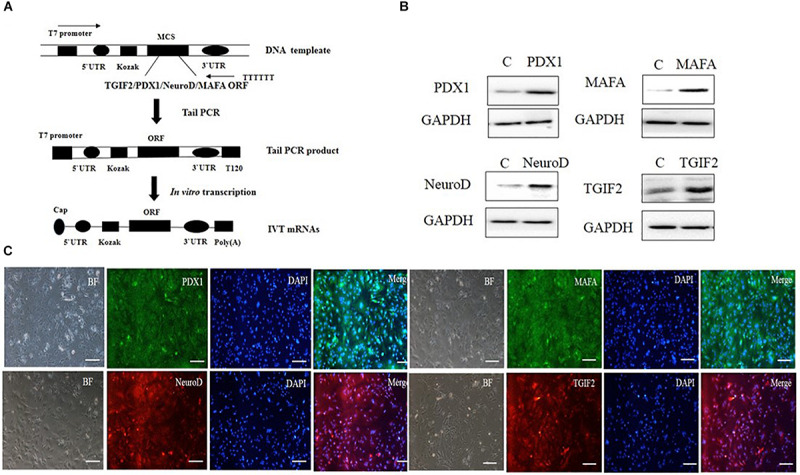
*In vitro* synthesis and identification of mRNAs. **(A)** Schematic diagram of different kinds of RNA synthesis *in vitro*. **(B)** The four kinds of IVT mRNAs were transfected into hepatocytes, and 12 h later, the cells were evaluated for protein expression by western blotting. **(C)** The four kinds of IVT mRNAs were transfected into hepatocytes, and 12 h later, protein expression was measured by immunocytochemistry (scale bar: 100 μm). BF, bright field.

ORFs encoding TGIF2, PDX1, NeuroD1, and Mafa were cloned from mouse islet cDNA by polymerase chain reaction (PCR). *In vitro* transcribed template construction and RNA synthesis are schematized in Figure 1A. mRNAs were synthesized with the use of the MEGAscript T7 kit (Ambion) according to the manufacturer’s instructions. The reaction used ARCA cap analog (New England Biolabs), 5-methylcytidine triphosphate instead of cytidine and pseudouridine triphosphate instead of uridine (TriLink Biotechnologies). Reactions were incubated for 5 h at 37°C followed by Antarctic Phosphatase (New England Biolabs) treatment for 2 h at 37°C to remove residual triphosphates. The synthesized RNA was purified with Ambion MEGAclear spin columns (Ambion) and quantified using a Nanodrop spectrophotometer (Thermo Fisher Scientific). mRNA transfection was carried out with TransIT-mRNA (Mirus). *In vitro* transcribed mRNAs were diluted in Opti-MEM basal media (Gibco), followed by the addition of boost reagent and TransIT-mRNA sequentially. After 2 min incubation at room temperature (RT), the RNA–lipid complexes were delivered to the culture medium. Four hours later, the medium was replaced with normal culture medium.

### Differentiation of Hepatocytes Into IPCs

The hepatocytes were infected with TGIF2, PDX1, NeuroD1, and Mafa mRNAs sequentially from day 1 to day 4, with each day assigned to one transcription factor. At day 6, the cells were harvested to assess transcription factor expression and insulin expression. For islet-like cluster formation, the cells differentiated for 6 days were dissociated and seeded in the well with gelatin (0.1%; Sigma) for another 3 days.

### Immunocytochemistry

The cells were fixed with 4% paraformaldehyde (Sigma) for 10 min and washed three times with PBS. The cells were permeabilized with 0.1% Triton-X 100 (Sigma) for 20 min and blocked with 2% bovine serum albumin at RT for 60 min. Then, the cells were incubated with rabbit anti-mouse insulin (1:100) and rabbit anti-mouse C-peptide (1:100) (Santa Cruz Biotechnology) antibodies for 60 min at 37°C. After rinsing with PBS three times, the cells were incubated with FITC-conjugated rabbit anti-mouse IgG 1:200 (Santa Cruz Biotechnology) secondary antibodies for 60 min, followed by rinsing three times with PBS. The cell nuclei were counterstained with DAPI. The cells were examined under a fluorescence microscope (Leica DMIRE2).

### Western Blot Analysis

Cells transfected with IVT mRNAs were washed with PBS three times and collected with cell lysis buffer. Cell lysates were incubated on ice for 30 min. Proteins in the cell lysates were separated by 12% sodium dodecyl sulfate-polyacrylamide gel electrophoresis and electrotransferred to nitrocellulose membranes. The blot was placed in blocking buffer for 1 h at RT, followed by incubation with a 1:500 dilution of rabbit anti-human TGIF2, PDX1, NeuroD1, Mafa, c-Jun N-terminal kinase (JNK), and p-JNK antibodies (Abcam) overnight at 4°C. The blots were rinsed with PBS with Tween-20 three times and incubated with mouse anti-rabbit horseradish peroxide-conjugated secondary antibody (1:1000) for 60 min, and bands were detected by means of chemiluminescence with ECL Hyper film.

### Quantitative RT-PCR

Total RNA was extracted using TRIzol (Ambion) following the manufacturer’s recommendations and quantified by UV spectroscopy. To prepare RNA for quantitative real-time PCR analysis, 2 μg of total RNA was converted to cDNA with the use of the Fast Quant reverse transcription kit with gDNase (TIANGEN). Quantitative real-time RT-PCR was performed using a standard SYBR Green PCR kit (Invitrogen) protocol on an ABI Step One Plus sequence Detection System (Applied Biosystems). Each sample was detected in triplicate. All the values were normalized to the reference genes and calculated using the software REST. Statistical significance in qPCR experiments was calculated using the REST randomization test. The primer sets used in this study are listed in Table 1.

**TABLE 1:** The primers for qRT-PCR.

Gene name	Sequence	Size (bp)
Afp F	CGAGGAGTGTTGCCAAGGAAA	141
Afp R	CAGAAGCCTAGTTGGATCATG	
Alb F	TGCTGCTGATTTTGTTGAGG	169
Alb R	GCAGCACTTTTCCAGAGTGG	
Hnf4a F	AACCACGCTACTTGCCTTTGCT	104
Hnf4a R	TCTGATGGGACACAGCCTACTTCT	
Hex F	GAGGTTCTCCAACGACCAGA	202
Hex R	GTCCAACGCATCCTTTTTGT	
Lgr5 F	CAGTGTTGTGCATTTGGGGG	136
Lgr5 R	CAAGGTCCCGCTCATCTTGA	
Cdx2 F	AAACCTGTGCGAGTGGATG	221
Cdx2 R	CTGCGGTTCTGAAACCAAAT	
Foxa2 F	CATCCGACTGGAGCAGCTA	178
Foxa2 R	GCGCCCACATAGGATGAC	
Neurod F	AAGGCAAGGTGTCCCGAGGC	109
Neurod R	CATCAGCCCGCTCTCGCTGT	
Pdx1 F	CCACCAAAGCTCACGCGTGGA	156
Pdx1 R	GGCGGGGCCGGGAGATGTATT	
Tgif2 F	CTATCTGCACCGCTACAACG	107
Tgif2 R	GGGCATTGATGAACCAGTTAC	
Isl1 F	GCGGCAATCAGATTCACGT	181
Isl1 R	GCGCATTTGATCCCGTACAA	
Mafa F	CTTCAGCAAGGAGGAGGTCA	195
Mafa R	TTGTACAGGTCCCGCTCTTT	
NKX6.1 F	ACCTTTGGGCTCACATAACCC	120
NKX6.1 R	AGGATGTCGTTGATGCCGTG	
NKX2.2 F	GAGGGCCTCCAATACTCCCT	105
NKX2.2 R	GTCATTGTCCGGTGACTCGT	
Vangl F	CCCCGAGTCTTCGTGTTACG	89
Vangl R	AAGATGCGCACACCGTAGAA	
Celsr F	GAGGCCATCACCAACTTCCC	157
Celsr R	TTACCAGCTCTACCCAAACGG	
Tcf7 F	GGAGCTGCAGCCATATGATAGA	205
Tcf7 R	AGATAGTGCATGCCACCTGC	
Tle3 F	GAAGTCAAGCTCACTTGGCG	92
Tle3 R	TGACACGGAATTGTTCGTGC	
Camk2b F	GTTTGGATTTGCGGGAACGC	102
Camk2b R	TACAGGATCACCCCACATGC	

### Detection of Insulin Secretion

After differentiation, the cells were washed five times with PBS and incubated for 1 h in Krebs-Ringer bicarbonate buffer (120 mmol/L NaCl, 2.5 mmol/L CaCl_2_, 1.1 mmol/L MgCl_2_, 25 mmol/L NaHCO_3_, 0.1% bovine serum albumin) containing 5.5 mmol/L glucose. The cells were washed again and incubated in Krebs-Ringer bicarbonate buffer containing 25 mmol/L glucose for an additional 1 h. Supernatants from cells stimulated with 5.5 or 25 mmol/L glucose were collected and analyzed with a human insulin enzyme-linked immunosorbent assay (ELISA) kit (Millipore) according to the manufacturer’s instructions.

### Dithizone Staining

The differentiated cell clusters on the gelatin coated plate were washed with PBS three times, and 10 μL of the dithizone stock (10 mg/mL) was then added to 1 mL of the culture medium. Cells were incubated at 37°C for 15 min and rinsed three times with PBS. Culture dishes were refilled with differentiation medium, and clusters were observed with the use of an inverted light microscope.

### Cellular Insulin Content

Insulin was extracted by the acid/ethanol method (Hamid et al., 2002). The approximately 2.5 × 10^5^ cells per well were extracted by incubation overnight at 4°C with 500 μL of acid/ethanol solution (1.5% HCl, 75% ethanol, 23.5% H_2_O), homogenized and incubated overnight with rotation. Then homogenized samples were centrifuged at 2000 rpm (15min, 4°C) and supernatant was nertralized with 1 mol/L Tris-Cl buffer (PH7.4). The insulin levels were measured using the ELISA kit and the results were presented as means ± SD.

### Statistical Analysis

The results are presented as the mean ± SD. The statistical significance of differences was tested using Student’s *t*-test in Microsoft Office Excel. Values of *p* < 0.05 were considered statistically significant.

## Results

### Efficient Transfection of Hepatocytes With IVT mRNAs

*In vitro* transcribed mRNA is a useful tool in gene delivery. It has higher safety than other approaches due to the avoidance of genomic insertion, and it can be translated efficiently in cells. The synthesis of mRNAs in our study is illustrated in a flow chart (Figure 1A). According to the flow chart, we synthesized four kinds of mRNAs: TGIF2, Pdx1, NeuroD1, and Mafa. To confirm that the IVT mRNAs could enter the hepatocytes and subsequently be translated into protein, the four IVT mRNAs were transfected into the liver cells. After 12 h, the cells were harvested to detect protein expression through immunostaining and western blotting. The results showed that all four kinds of IVT mRNAs entered the hepatocytes efficiently by a cationic vehicle and were translated into proteins properly (Figures 1B,C). The use of IVT mRNAs provides the possibility of controlled and temporary delivery of the desired transcripts to induce protein expression. This method is safe for use in gene therapy and will likely receive better clinical acceptance in the future.

### Morphological Characteristics and Gene Expression During Transdifferentiation of Hepatocytes Into IPCs

**FIGURE 2:**
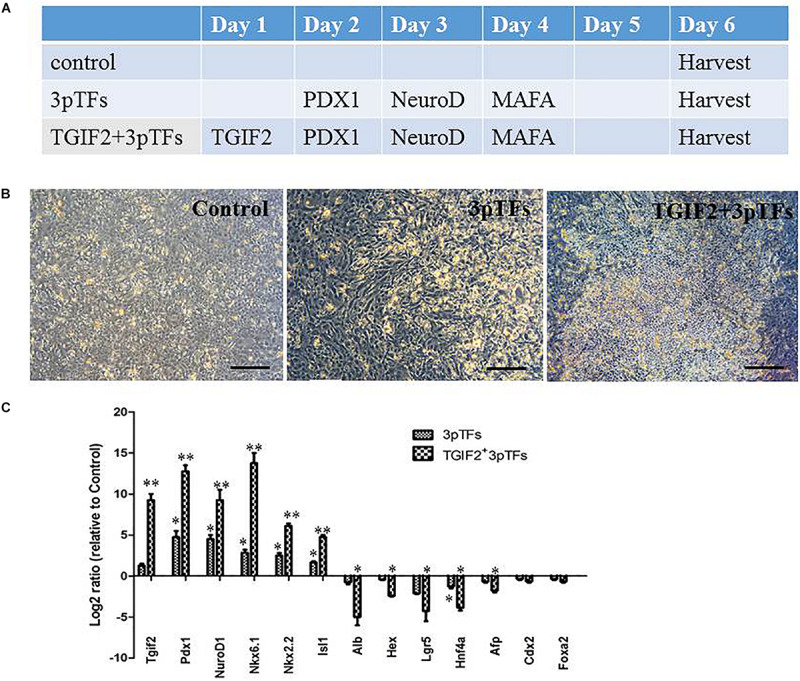
Morphological changes and gene expression during transdifferentiation of hepatocytes into IPCs. **(A)** The hepatocyte transdifferentiation protocol for obtaining IPCs with IVT mRNAs. **(B)** Changes in the morphology of hepatocytes on day 6 of differentiation (scale bar: 250 μm). **(C)** RT-qPCR analysis of pancreatic gene expression and hepatic gene expression on day 6 after transdifferentiation. The data were normalized and represented as the log2 expression ratio between transduced and control cells. Values shown are the mean ± SEM (*n* = 3) *p < 0.05.

Many studies have suggested that the simultaneous expression of several pTFs increases the efficiency of transdifferentiation compared with that of transdifferentiation induced by individual pTFs. We first started the transdifferentiation of hepatocytes with a modified protocol reported by Dana et al. (2014). Their experimental results suggested that transcription factor-induced transdifferentiation from liver to pancreas is a progressive and hierarchical process. To analyze the effect of TGIF2 during transdifferentiation, we compared the effects of three major pTFs, PDX1, NeuroD1, and Mafa, to those of TGIF2 plus these three pTFs on hepatocyte to IPC transdifferentiation. Cultured hepatocytes were infected with TGIF2, Pdx1, NeruoD1 and Mafa mRNA in a stepwise manner, as illustrated in Figure 2A. The cell morphology changed rapidly, and the cells began to coagulate 6 days after differentiation. The cells that were first transduced with TGIF2 changed more obviously than the cells that were transduced with only the mRNAs of the three pTFs (Figure 2B). To determine the changes in transcription factor expression during the differentiation period, the expression of liver and pTFs was assayed by qRT-PCR at different times after stepwise transfection with IVT mRNAs. Ectopically transduced Tgif2 mRNA strongly inhibited hepatic gene expression and initiated a set of pancreatic genes. Importantly and as expected, the levels of pancreatic transcripts were higher in cells transfected with all four mRNAs than in those transfected with the three pTFs alone (Figure 2C).

### Sequential Transduction With IVT mRNAs of TGIF2 and Three pTFs Increases the Efficiency of Hepatocyte Transdifferentiation Into IPCs

**FIGURE 3:**
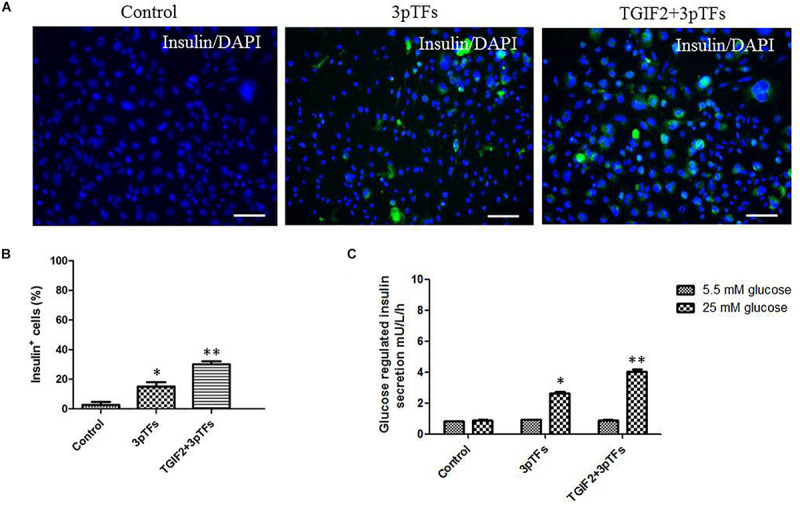
Detection of insulin expression in differentiated cells and their response to glucose. **(A)** Immunofluorescence staining of treated hepatocytes for insulin (scale bar: 100 μm). **(B)** The percentage of insulin-positive cells was calculated by counting at least 1000 positive cells from at least two independent experiments. **(C)** Detection of insulin release in response to glucose by ELISA. Data are presented as the mean ± SD of three independent experiments (*p < 0.05 and **p < 0.001).

Immunocytochemistry was performed to test for insulin-positive cells after differentiation (Figure 3A). The group induced with TGIF2 plus the three pTFs showed an increase in the number of IPCs by 30%, while the group induced with the three pTFs alone showed an increase of only 15% (Figure 3B). Secretion of insulin was measured after the cells were stimulated by glucose. Insulin secretion by IPCs from the groups induced with TGIF2 plus the three pTFs or the three pTFs alone was measured by ELISA. As shown in Figure 3C, the IPCs derived via these methods secreted less insulin in the medium with a lower glucose concentration (5.5 mM) and more insulin in the medium with a higher glucose concentration (25 mM). Additionally, the IPCs derived from group treated with TGIF2 plus the three pTFs had higher insulin secretion levels than the IPCs derived from the group treated with the three pTFs alone.

### Islet-Like Cluster Formation

**FIGURE 4:**
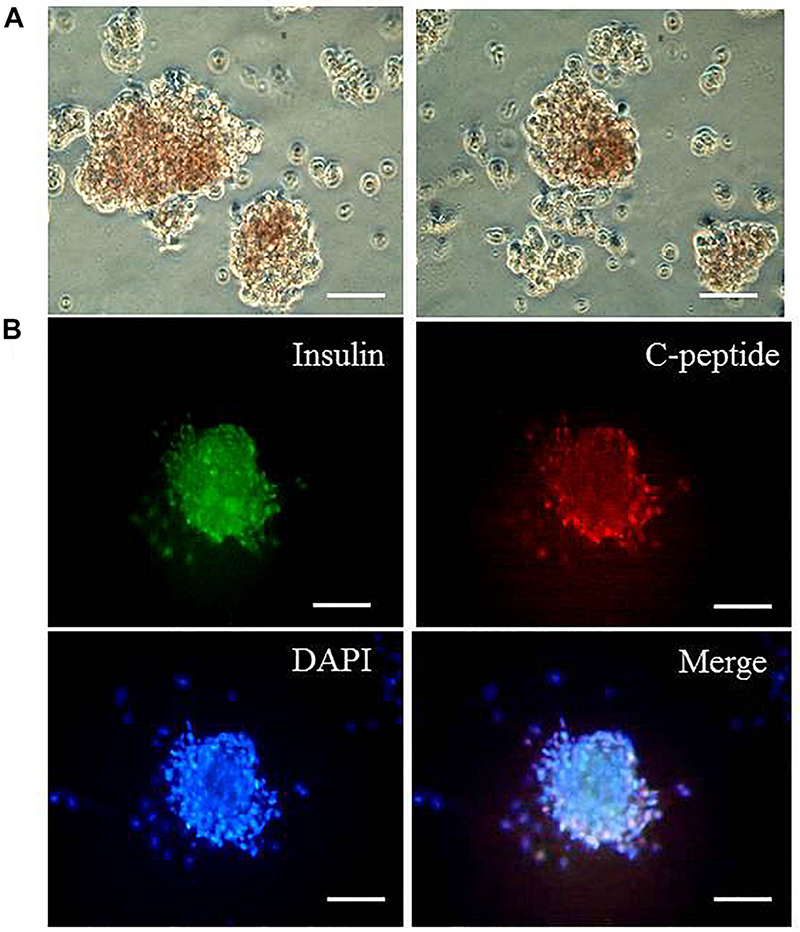
Islet-like cluster formation. **(A)** Dithizone staining of differentiated cells that were induced with TGIF2 together with three pTFs and grown on gelatin for another 3 days (100 μm). The hepatocytes differentiated by the three pTFs failed to grow under the same conditions. **(B)** Detection of coexpression of insulin and C-peptide for islet-like clusters by immunostaining (scale bar: 100 μm).

We then tested whether the differentiated cells could form islet-like clusters. The cells that were differentiated for 6 days were plated in the well coated with gelatin for another 3 days. The differentiated cells derived from the addition of TGIF2 mRNA could form islet-like clusters. By contrast, control non-transduced hepatocytes and cells differentiated with the three pTFs did not form clusters under the same conditions. Dithizone staining was used to detect the expression of insulin in the islet-like cell clusters (Figure 4A). Immunocytochemistry was performed to test the expression of insulin and C-peptide proteins in the islet-like cell clusters and determined that the clusters were insulin and C-peptide positive (Figure 4B). This indicates that TGIF2 is important for control islet-like cluster formation. The cellular insulin content of the islet-like clusters was 6.43 ± 0.09 IU/L.

### Tgif2 Induces Transdifferentiation More Efficiently by Remodeling the Expression of Genes in the Wnt/PCP Pathway

**FIGURE 5:**
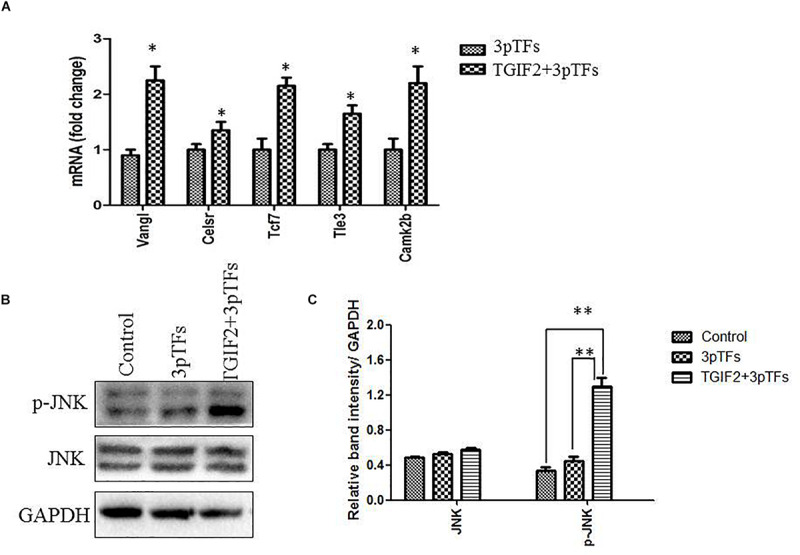
Changes in the Wnt/PCP signaling pathway during the transdifferentiation of hepatocytes to IPCs. **(A)** Detection of expression of genes in the Wnt/PCP pathway compared to that in the control. Data are presented as the mean ± SD of three independent experiments (*p < 0.05). **(B)** Detection of JNK or phosphorylated JNK protein by western blotting. **(C)** The relative densities of JNK and phosphorylated JNK protein bands were normalized to actin in the same samples. Data are presented as the mean ± SD of three independent experiments (**p < 0.001).

WNT signaling can endow cells with plasticity, which allows them to change their transcriptional program and developmental fate when transfected with ectopic transcription factors. It has been shown that the Wnt/PCP signaling pathway is crucial for directing endodermal progenitors toward the liver or islets during the process of embryonic development. To study the effect of TGIF2 during differentiation, the cells were collected to analyze the expression of genes in the Wnt/PCP signaling pathway one day after transfection of TGIF2 mRNA. Our results showed that TGIF2 overexpression in hepatocytes can activate the expression of key genes controlling cell polarity and genes in the Wnt/PCP signaling pathway (Figure 5A). Many core Wnt/PCP cell polarity genes, such as Vangl and Celsr, as well as downstream Wnt transducers, such as Tcf7, Camk2b, and Tle3, were found to be upregulated in transfected cells. The western blot results showed that TGIF2 could increase the level of JNK protein phosphorylation, which played an important role in controlling the Wnt/PCP signaling pathway (Figures 5B,C). Our results showed that TGIF2 may promote efficient transdifferentiation of hepatocytes by changing cell polarity.

## Discussion

Developmentally, the liver and pancreas are closely related. They share a number of characteristics, including the ability to respond to glucose levels, and a large group of specific transcription factors. Thus, transconversion from liver to pancreas might be much easier than conversion to any other organ. To increase the efficiency of transdifferentiation, several research groups used simultaneous ectopic expression of three transcription factors, PDX1, NeuroD1, and Mafa, to mimic progressive pancreatic development. When ectopic transcription factors are added in a stepwise manner, the efficiency of transdifferentiation increases (Tang et al., 2013). However, delivery of transcriptional factors into hepatocytes using virus-mediated strategies results in immune-based hepatotoxicity, which is not suitable for clinical applications.

*In vitro* transcribed mRNAs can produce proteins without any risk of randomly integrating echogenic genes into the genome, and this is a very useful approach for regenerative medicine (Johler et al., 2015; Nevo-Shenker et al., 2019). *In vitro* transcribed mRNAs have been frequently used to produce induced pluripotent stem cells or transdifferentiating cells in a precisely controlled pattern. Previously, we reported a method for inducing human embryonic stem cells (hESC) to differentiate into IPCs by transfecting IVT mRNAs of transcription factors specific for pancreatic development (Wang et al., 2015). The limitations of transdifferentiation from hepatocytes to IPCs could be the lack of appropriate interaction partners or the presence of antagonistic factors in hepatocytes that lock the cell identity, hampering cell plasticity and conversion. Considering these lineage restrictions, we assumed that the developmental regulator TGIF2, a decision-making factor for pancreas versus liver fate, might be an effective reprogramming determinant to achieve transdifferentiation of hepatocytes into pancreatic islet cells. It has been found that overexpressing TGIF2 in hepatocytes could cause them to undergo extensive transcriptional remodeling, in which the original hepatic identity is suppressed and a pancreatic progenitor-like phenotype is induced (Spagnoli and Brivanlou, 2008; Wang et al., 2017; Espona-Noguera and Ciriza, 2019). Cells differentiated with three pTFs could not form islet-like clusters. Therefore, we used TGIF2 together with three pTFs to induce differentiation. The cells changed rapidly to round or oval shapes and began to gather together to form islet-like clusters after transfection of the four kinds of mRNAs in differentiation medium, and the clusters were insulin- and C-peptide-positive. It seems that before the cell identity switch, the reprogramming activity of TGIF2 supports a “lineage-restricted” dedifferentiation step.

In this study, we demonstrate that freshly isolated adult mouse hepatocytes can be cultured *in vitro* and induce transdifferentiation into IPCs. We focused on the combined introduction of IVT mRNAs of TGIF2 and three pTFs to assess their application for the transdifferentiation of hepatocytes into IPCs, thereby establishing an efficient, footprint-free, and simplified protocol to transdifferentiate hepatocytes into IPCs. The administration of TGIF2 mRNA combined with sequential addition of three pTF mRNAs could induce transdifferentiation more efficiently than sequential administration of the three ectopic pTFs alone. The transdifferentiated cells exhibited insulin production *in vitro*, especially when challenged with glucose.

The mechanism of TGIF2 function during transdifferentiation from hepatocytes to IPCs is the basis of cell-based therapy for T1D in clinical applications. Our results showed that TGIF2 could increase the level of JNK protein phosphorylation, and JNK protein played an important role in the signaling pathway. This provides a foundation for further studies on the role of JNK protein phosphorylation in the trans-differentiation of hepatocytes into insulin-secreting cells induced by TGIF2.

In summary, our work demonstrates that the protocol that used TGIF2 combined with the three pTFs could promote cell fate alteration and induce hepatocyte transdifferentiation toward IPCs more efficiently than the protocol that used the three pTFs alone. Through activating the expression of Wnt/PCP pathway-related genes, TGIF2 remodels cell fate. To bring transdifferentiation-based therapies closer to clinical application, it is necessary to further increase their efficacy and further reduce the number of transdifferentiated cells required. mRNA-based gene delivery can be precisely controlled via the expression level and timing. *In vitro* transcribed mRNAs are useful for highly efficient gene manipulation in hepatocytes, and differentiated cells have great potential for therapeutic applications. However, further investigation is required to transplant differentiated cells into the renal capsule of diabetic mice. The technology of mRNA-based gene delivery can also be utilized as a useful tool to deliver genes *in vivo*, but the mRNA should be wrapped by biodegradable nanomaterials to further increase its stability. It is very safe for gene therapy use and will likely receive better clinical acceptance in the future.

## Conclusion

We established an efficient method to genetically manipulate hepatocytes by IVT mRNAs encoding three pTFs, PDX1, NeuroD1, and Mafa together with TGIF2. We were able to successfully establish a rapid and footprint-free protocol for trans-differentiation of hepatocytes into hepatocytes into IPCs. Since it is footprint-free and integration-free to the host genome. Thus, our method may be well suited for future regenerative therapies for diabetes.

## Data Availability Statement

All datasets generated for this study are included in the article/supplementary material.

## Author Contributions

SM, MY, and WZ contributed to the data curation, investigation, and methodology. LD contributed to the writing—review and editing. YD, XG, YY, and JT contributed to verifying all the experimental results. DL and XW contributed to the writing—original draft and funding acquisition.

## Conflict of Interest

The authors declare that the research was conducted in the absence of any commercial or financial relationships that could be construed as a potential conflict of interest.
